# Cork Façades as an Innovative and Sustainable Approach in Architecture: A Review of Cork Materials, Properties and Case Studies

**DOI:** 10.3390/ma17174414

**Published:** 2024-09-07

**Authors:** Isabel Miranda, Helena Pereira

**Affiliations:** Centro de Estudos Florestais, Laboratório Associado TERRA, Instituto Superior de Agronomia, Universidade de Lisboa, 1349-017 Lisboa, Portugal; imiranda@isa.ulisboa.pt

**Keywords:** building, expanded corkboards, construction, agglomerated cork, cork composites

## Abstract

Façades give the first impression of a structure, reflecting the overall aesthetic appeal, architectural styles, cultural influences, and technological advancements. Emphasis on sustainability is increasing, with a shift towards eco-friendly and energy-saving materials, triggered by decreasing the environmental impact of construction. Cork is a green competitive material for various engineering and design applications due to its biological formation, sustainable production and a portfolio of properties including low density, impermeability, viscoelastic behaviour and high thermal insulation that derive from its cellular and chemical features. This work presents cork materials used in building façades and their properties, also giving information on cork production and processing into cork-based products as a review of the existing published research, while also identifying knowledge gaps and further research needed. Historical examples of cladding of constructions with raw cork are given, while the contemporary innovative use of cork façades was triggered by some designs of well-known architects with outdoor application of expanded cork agglomerates. Examples of different historical and contemporary constructions were assembled and critically assessed by the authors. The aim is to give integrated information of cork as a natural, renewable and sustainable material to raise the interest of designers, architects and engineers to explore cork, blending aesthetics with environmental responsibility, targeting a more sustainable and resilient built environment.

## 1. Introduction

Façades act as the face of architecture in buildings, providing the first impression of a structure, reflecting architectural styles, cultural influences, and technological advancements, while influencing the overall aesthetic appeal. A modern façade design may generate a unique appearance for the building, sometimes becoming a well-known public landmark.

Façade systems comprise structural elements and the building envelope that provides weather resistance and thermal, acoustic and fire-resisting properties. They are multifunctional and highly adaptive architectural systems, acting as a physical and protective barrier, a mediator between indoor and outdoor environments, combining structural requirements with energy efficiency and comfort needs [1,2]. The materials chosen for building façades play a pivotal role in determining their durability, aesthetic appeal, and overall performance. Traditional materials like brick, stone, and concrete have been extensively used due to their strength and longevity, but contemporary architecture explores a diverse range of materials, including glass, steel, aluminum and composites of different types.

In recent years, there has been a growing emphasis on sustainability, leading to a shift towards eco-friendly and energy-saving materials, driven by the recognition of the environmental impact of construction and the imperative to create more energy-efficient structures, thereby calling attention to the insulation properties of the materials [3,4,5]. Eco-friendly materials include recycled and reclaimed elements, thereby reducing the demand for new resources and contributing to waste reduction, while energy-saving materials aim to improve the thermal performance of buildings and reduce energy consumption. This trend has led to integration of solar panels, high-performance insulation, and green façade systems, which incorporate living plants into the building envelope.

An exciting addition to the palette of sustainable materials for building façades is the biomaterial cork. Cork, obtained from the bark of cork oak trees, is a renewable cellular material that possesses remarkable properties while being the product of a sustainable tree exploitation within a highly valued environmental ecosystem [6]. Cork oak forests are one of the best examples of a balanced ecosystem of conservation and socio-economic development using multifunctional agro-forestry systems, which combine cork production with cattle grazing, hunting, other non-wood productions and provision of extensive ecosystem services [6].

Cork is a competitive material for various engineering and design applications in buildings and infrastructures due to a portfolio of properties including low density, impermeability, viscoelastic behaviour and high thermal insulation that derive from its cellular and chemical features [7,8]. Among its properties, cork is well known for its natural insulation capabilities, providing thermal resistance and contributing to the energy efficiency of buildings by regulating internal temperatures [9,10]. Therefore, it has been used as an internal insulation in walls and roofs, mostly in the form of expanded cork agglomerate boards or granulates, as a natural alternative to glass wool, stone wool or organic foamy materials [11,12,13,14,15]. It is also used in External Thermal Insulation Composite Systems (ETICS), where it shows good structural and insulation performance [16]. Cork composites are mostly applied as surfacing and finishes for interior walls and pavements [7,17,18].

The application of cork in façade systems as an external cladding has been little addressed, although singular experiments have been made with high public impact. One of the first examples is the Portuguese Pavilion in the World Exhibition of Hannover in 2000, which had façades covered by expanded cork insulation boards whose texture and dark colour conferred a singular architectural appeal. A subsequent example was the Portuguese Pavilion in the 2010 World Expo, Shanghai, China, a massive polyhedral volume totally clad by expanded cork insulation boards. These and other examples will be detailed further on. Such cork façades offer a distinctive aesthetic appeal with a warm and organic texture while offering functional benefits e.g., its lightweight nature makes it easy to work with, reducing the overall load on the building structure, and is resistant to moisture and decay, enhancing the longevity and durability of the façade.

There is a large array of research on cork and cork products, as synthetized in the reference book of Pereira [6], and specifically on cork as a building material, as reviewed in Knapic et al. [7] as well as in several technical manuals on cork [19,20]. However, there is a lack of comprehensive work targeting cork application as cladding of building façades. This paper presents an overview of cork materials used in building façades with a summary of their properties as a review of the existing published research by the cited authors, while also making an original identification of knowledge gaps and studies proposed to add further knowledge, for an enhanced use of cork in cork façades and other external uses. Historical cladding of constructions with raw cork is given, while the contemporary innovative use of cork façades was triggered by some designs of well-known architects’ outdoor application of expanded cork agglomerates. Examples of different historical and contemporary constructions with cork façades are assembled and critically assessed by the authors. First, information is given briefly on cork, the material, including its biological formation and production, as well as the cellular and chemical features and properties in a brief and comprehensive way, while references to published research are provided if a more exhaustive approach is intended. The processing of the raw material cork into cork-based products is summarized next, with details of cork composites and expanded cork agglomerates and their specific properties as given by the published studies. A synthesis of cork properties of interest for building façades is made, following original critical reasoning, while knowledge gaps are identified, and prospective research studies are proposed. The use of cork in the cladding of buildings is presented with an historic overview and recent examples of built constructions as assembled by the authors. The aim is to provide integrated information of cork as a natural, renewable and sustainable material to raise the interest of designers, architects and engineers to explore cork and blend aesthetics with environmental responsibility, thereby contributing to a more sustainable and resilient future for the built environment.

## 2. Cork Production and Properties

### 2.1. Cork Production

Cork is the external layer of the periderm in the bark that covers tree stems and branches, functioning as a protective layer, as it is well described in plant anatomy [21]. While cork is present in the periderms of all species, it usually is limited to a few cell layers, and only some species have periderms with a substantial cork layer, the so-called cork-rich barks [22]. The cork that has been used since antiquity and has provided the raw material to a dedicated industry that has developed along the last two centuries comes from the cork oak (*Quercus suber* L.), an evergreen oak species. The cork oak originated in the western Mediterranean basin and now has spread to forests in southern Europe in Portugal and Spain, and in restricted areas of France and Italy, and in North African Morocco, Algeria and Tunisia [6]. 

Cork oak forests, known as *montados* in Portugal, *dehesas* in Spain and *azaghar* in Morocco, cover a total area of approximately 2.1 million hectares [23]. Their management is oriented towards cork production, but they are exploited as a low-impact agro-forestry system, with high biodiversity and conservation value [24]. Every year, approximately 200 thousand tonnes of cork are extracted from the cork oak forests, the majority concentrated in Portugal and Spain, with these two countries providing 80% of the worldwide cork production [23].

The cork formation in the cork oak has characteristics that allow a sustainable exploitation by following a specific silviculture known as subericulture, which is well established and regulated. The process of cork formation and of cork oak exploitation is well detailed in Pereira [6], and here only briefly described. The cork forms a continuous envelope around the stem, growing in thickness by about 1–3 mm each year, and it can be pulled out without damaging the underlying inner bark and wood cambium when the tree is physiological active in spring and early summer. After extraction of the cork, the tree regenerates a new periderm in the external part of the bark that will form a new cork layer with similar characteristics. The process may be repeated with successive cork extractions. This ability to develop a new periderm each time is the basis for the sustainability of cork production and cork oak exploitation. In the present management of cork oak forests, cork extraction is performed at a tree age of about 25 years, and then with intervals of 9 years or more, for up to a tree age of approximately 150 years. 

**Figure 1:**
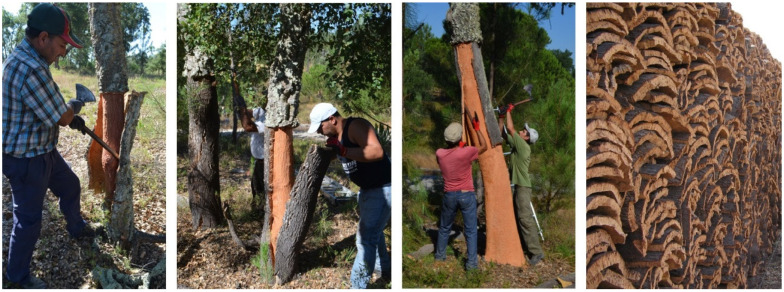
Photographs, from left to right, of the cork stripping of a juvenile cork oak tree with virgin cork, of a young cork oak with second cork, and of a mature cork oak under cork exploitation with reproduction cork, as well as a field pile of reproduction cork planks. Photos by Helena Pereira.

The operation of cork stripping is done manually by cutting large rectangular planks with an axe and pulling them out. The cork in the first periderm is called virgin cork, and shows numerous and deep cracks that run mostly longitudinally due to the radial enlargement of the juvenile tree. The following cork extraction yields the cork produced by the second periderm (called second cork) that also has longitudinal fissures due to the still high tangential growth stress of the young tree. When the second cork is removed, the new cork layers (called reproduction cork) no longer fissure since the tangential growth stress is much smaller. Figure 1 shows the cork stripping of a juvenile cork oak covered with virgin cork, of a young tree with second cork and of a mature tree undergoing the removal of reproduction cork, as well as a pile of cork planks.

Another cork-rich oak species has seen a growing commercial interest in its cork production. It is the Chinese cork oak (*Quercus variabilis* L.), native to East Asia, including China, the Korean Peninsula, Japan, Laos and Thailand [22,25]. The cork production in China and South Korea was at a limited scale until recently, but increased in the last few years due to manufacturing and marketing of cork products [26]. The subericulture of *Q. variabilis* is still in its first stages, and the cork is directed to the production of granulates, with products based on granulated cork agglomerates that are overall of less quality and market value than those from *Q. suber* [27].

### 2.2. Cork Structure

Cork belongs to cellular materials of biological origin of a closed cell foam type. Cork has a regular and compact cellular structure arrangement of small closed thin-walled cells. The individual cells are hexagonal prisms that are packed base-to-base in columns parallel to the radial direction in the tree in a compact structure without intercellular voids [6]. When observed in the transverse section (the plane perpendicular to the cork oak axis) or in the radial section, the structure is of a brick-wall type, with the cells appearing with a rectangular form; in the tangential section (the plane perpendicular to a stem diameter), the cork cells appear polygonal, mostly as hexagons with a honeycomb structure [28]. Cork has small and thin-walled cells (prism height 30–40 µm, base edge 13–15 µm, wall thickness 1–1.5 µm) and contains about 4 × 10^−7^ to 7 × 10^−7^ cells per cm^3^, representing a solid fraction under 20% of the total volume [6]. The structure is anisotropic, but cork can be considered a transversally isotropic material because the radial direction is a symmetry direction [29]. 

**Figure 2:**
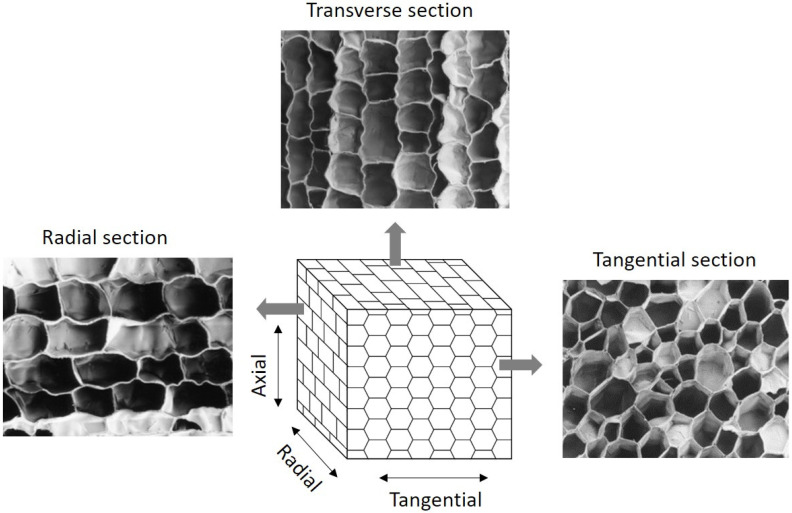
Schematic representation of the 3D cellular structure of cork and structure of cork as observed by scanning electron microscopy in the three main sections: tangential section (perpendicular to the tree’s radial direction), transverse section (perpendicular to the tree’s axial direction), and radial section (the tree’s radial section). Adapted from Pereira [8] and Oliveira et al. [30].

Figure 2 shows scanning electron micrographs of the cork cells in the three sections (tangential, transverse and radial) and a 3D schematic representation of the cork tissue.

The cork mass itself is uniform and devoid of cell-type distinction, although dimensional differences are present, particularly regarding the cells formed at the end of the growing season that are radially shorter and have thicker cell walls, thereby allowing us to distinguish annual rings in cork. Lenticular channels, arising from the so-called periderm lenticels, cross cork along the radial direction, and constitute a heterogeneous microporosity of natural origin, and are a distinctive feature of cork. Their role is to enable gas exchange of the inner tissues with the outside environment [6].

### 2.3. Cork Chemistry

Most of the characteristics of cork are explained by the features of its structure and by the cell wall chemical composition [6,8]. The chemical composition of cork cell walls is dominated by a biopolymer known as suberin, which accounts for about 45% of the cork’s mass and acts as a major structural component. Suberin is specific to cork (commonly referred to as a subereous tissue). Suberin is a polyester polymer, based on glycerol and long-chain hydroxycarboxylic acids, dicarboxylic acids and carboxylic acids, mostly with 16 and 18 carbons, including also ferulic acid and eventually other phenolic components [31]. The polymer develops by ester bonding between glycerol and long-chain acids and by their inter-monomeric bonding, leading to a macromolecular structure that develops three-dimensionally with a loose spatial spreading. Some of the conspicuous properties of cork may be directly related to the suberin chemical features, such as its hydrophobicity, low permeability and mechanical properties, namely its compressive behaviour. 

Lignin is the second most important structural polymer of cork cell walls, accounting for 25% on average. Lignin has a phenolic nature made up of the linking of phenylpropane monomeric units and develops as a more spatially compact structure that associates to suberin in the cork cell wall. Lignin is a well-known polymer because it is present in wood, responsible for rigidity and compressive resistance, while also adding to hydrophobicity. It is the association of suberin and lignin (their average ratio is 2.1) that confers cork its known properties.

The cork cell walls include the polysaccharides cellulose and hemicelluloses, which account for 15% on average and are therefore less important for defining cork properties. Cork also contains non-structural components, the extractives, that include non-polar and polar compounds (on average, 5.8% and 10.4%, respectively) that contribute to the hydrophobicity, low permeability and biological resistance of cork. 

### 2.4. Cork Properties

Table 1 is a summary of the range of values reported for the main properties of cork of interest for its main applications [6,32,33].

Cork is a light material with density values ranging from below 120 to over 200 kgm^−3^ [8]. The material’s lightness is the direct result of its cellular structure of closed thin-walled and air-filled cells, making up a material with a small solid fraction.

Cork has very low heat transfer properties explained by the large air volume contained in the small and closed cells that eliminate gas convection, with radiation being reduced by absorption in the numerous cells, and low conduction, given the small solid fraction of cork. The heat conductivity (λ) and the thermal diffusivity (α) of cork with a density 140–170 kg m^−3^ are 0.040–0.045 W m^−1^ K^−1^ and 1 × 10^−7^–1.5 × 10^−7^ m^−2^ s^−1^, respectively [32]. The sound transmission of the cork is very low, with an acoustic impedance of 1.2 × 10^5^ kg m^−2^ s^−1^ for a 120–200 kg m^−3^ natural cork [6,33].

Cork absorbs water very slowly, and water diffusion in cork is a considerably slow process, with diffusion coefficients of 1 × 10^−11^ and 4 × 10^−10^ m^2^ s^−1^ in the non-radial and radial directions, respectively [34]. The permeability of cork to liquid water is 280.5 × 10^−13^ mol m^−1^ s^−1^ Pa^−1^, and to water vapor, 110.1 × 10^−13^ mol m^−1^ s^−1^ Pa^−1^ [35]. Such permeation properties are the result of the cellular structure of cork without intercellular communication channels as well as of the chemical composition, namely the presence of the hydrophobic suberin and of the lipophilic extractives [36]. The permeability of cork to non-condensable gases, for example helium, oxygen, nitrogen and carbon dioxide, is also low [37,38].

Cork is a viscoelastic material that allows large deformations under compression without fracture, and with substantial dimensional recovery when stress is relieved [39]. The flexibility of suberin allows the cell wall folding without fracturing and the subsequent unfolding upon stress relief. Cork shows compressive stress–strain curves typical for cellular materials, with an approximately elastic region up to a strain of approximately 5%, followed by a large plateau, with a small positive slope for strains between about 5–70% strain caused by progressive buckling of cell walls, and a final steep stress increase for higher strains, corresponding to cell collapse. The elastic modulus (Young’s modulus, E) is between 10 MPa and 20 MPa, and the absorbed energy per unit volume (M) for the maximal load (ε_max_, 69.9–100%) for the radial compression is, on average, 2.4 J cm^−3^ [40,41,42].

The stress–strain curves for bending up to fracture show an initial linear elastic region until approximately a strain (ε_f_) of 2% and a stress (σ_f_) of 0.3 MPa, corresponding to a mean elastic modulus (Young’s modulus, E) of 14 MPa for the tangential direction and 21 MPa for the axial direction. This is followed by a region of decreasing slope corresponding to a gradual cork yielding up to a peak load, after which a near complete failure occurs in the zone under the neutral line in tension at an average fracture stress (σ_f_) of 1.2 MPa and a fracture strain (ε_f_) of 14% [43].

Regarding surface properties, cork is a hydrophobic material with low wettability towards polar liquids (e.g., water), and high affinity for non-polar liquids (e.g., non-polar resins), with a surface energy of 18 mN m^−1^ [44]. The contact angle of water on cork is between 84° and 100° [44,45].

Cork is thermally stable below 200 °C, and higher temperatures are required for significant chemical degradation, e.g., at 300 °C and above. The cork cell wall components have different thermal stabilities, with lignin and suberin being the most resistant [46].

Regarding its reaction to fire, the classification of natural cork according to the euroclass classification (EN 13501-1) is not well established and data are missing [7,47]. Flammability tests with cork samples showed that the flaming combustion of cork is slower with lower consumption of cork than that of other forest fuels, and has the following parameters: time-to-ignition (tTI, 89 s), flame duration (FD, 2006 s), peak heat release rate (PHRR, 208 kW m^−2^), average heat release rate (HRR, 65 kW m^−2^), total heat release (THR, 126 MJ m^−2^), mass loss rate (MLR, 0.024 g s^−1^), average effective heat of combustion (AEHC, 28.21 MJ kg^−1^) and residual mass fraction (RMF, 34%). These values suggest a higher fire resistance of cork than that of other types of tree barks [47].

When subject to weathering, cork undergoes surface colour alteration while maintaining its physical structure and cellular features. Very recent studies showed that the original light brown colour of cork (L* 55.7, a* 12.9, b* 23.5) changed to a lighter whitish colour (L* 58.3, a* 5.6, b* 15.2) after one year of outdoor exposure, mainly due to the photooxidation of lignin, in a process similar to what is known for wood, and affecting only the external cellular layer [48]. Therefore, the outdoor application of cork does not negatively impact its performance, with only the colour dynamics to be taken into consideration.

**Table 1:** Properties of cork, as reported by different authors.

Property	Value	References
Density (kg m^−3^)	120–180 (reproduction cork) 160–240 (virgin cork)	[6]
Thermal conductivity (W m^−1^ K^−1^)	0.045	[49]
Specific heat (J kg^−1^ K^−1^)	350	[32]
Acoustic resistivity (kg m^2^ s^−1^)	1.2 × 10^5^	[7]
Electrical conductivity (S m^−1^)	1.26 × 10^−10^ (25 °C) 1.67 × 10^−13^ (50 °C)	[50,51]
Permeability (mol m^−1^ s^−1^ Pa^−1^)	280.5 × 10^−13^ (liquid water) 110.1 × 10^−13^ (water vapor)	[35]
Water diffusion coefficient (m^2^ s^−1^)	1.0 × 10^−11^ (radial direction) 4.0 × 10^−10^ (non-radial direction)	[32]
Friction coefficient (cork/cork), boiled	0.97 (radial direction) 0.77 (non-radial direction)	[52]
Young’s modulus in compression (MPa)	10.4 (radial direction) 9.2 (non-radial direction)	[30,41]
Strain for the maximal load in compression (ε_max_, %)	83.4 (radial direction) 83.8 (non-radial direction)	[30,41]
Energy absorbed in compression per unit volume at that strain (J cm^−3^)	2.4 (radial direction) 2.2 (non-radial direction)	[30,41]
Young’s modulus in bending (MPa)	14 (tangential direction) 21 (axial direction)	[43]
Fracture stress in bending (MPa)	1.1 (tangential direction) 1.5 (axial direction)	[43]
Fracture strain in bending (%)	13.5 (tangential direction) 15.9 (axial direction)	[43]

## 3. Cork Products

### 3.1. Production of Cork Materials

**Figure 3:**
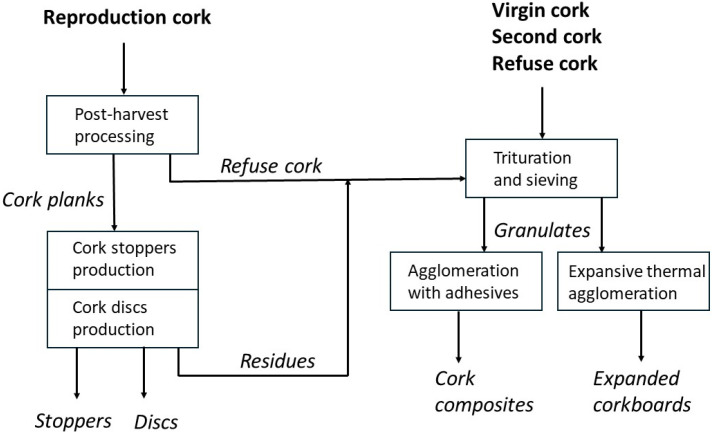
Schematic diagram of cork industrial processing and main products obtained.

The primary economic target of cork production is the manufacture of bottle closures, mainly wine natural cork stoppers and, to a lesser extent, cork discs to be applied onto composite stoppers for sparkling wines. A full resource use is adopted by the industry, and all residual materials from the production of cork closures, which represent as much as 75% of the raw material [6], as well as the cork raw materials not suitable for that production (virgin and second cork, refuse cork) are triturated and processed into agglomerated cork products. Figure 3 schematically shows the overall cork industrial processing and the main cork products that are obtained.

Cork agglomerates are classified into two types: (i) composite agglomerates, also called white agglomerates or just cork agglomerates; and (ii) pure or expanded agglomerates, also called insulation corkboards or black agglomerates. Cork composites are mainly applied as surfacing material for wall and floor coverings, and as bottle sealants as agglomerated cork stoppers. The expanded cork agglomerates are used as insulation materials mainly for thermal, acoustic and vibrational applications. A small description of their production processes, as well as of their properties, is given in sequence.

### 3.2. Cork Composites

**Figure 4:**
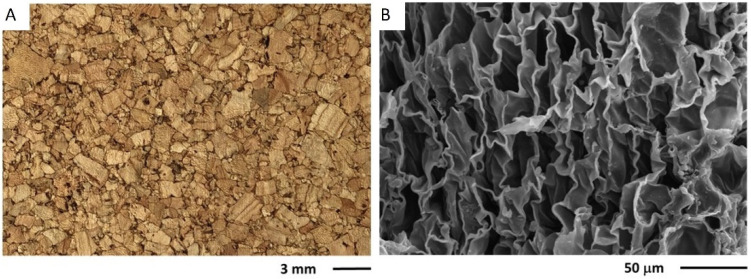
Visual aspects of a cork composite with 1–3 mm granules (**A**) as well as its cellular structure observed by scanning electron microscopy (**B**). Photos by Helena Pereira.

Cork agglomerates are made of cork granules that may vary from 1 mm to >1 cm mixed with a binder adhesive and other additives. The macroscopic aspects of these agglomerates are exemplified in Figure 4A, showing that the granules are pressed together into a rather compact structure, i.e., without significant intergranular voids. The cellular features of the cork granules in the agglomerate are those of cork (Figure 2), although in some regions, the effect of the pressing is shown in higher corrugation and cell distortion (Figure 4B). The adhesives include thermosetting polymers, such as urea–formaldehyde, melamine or phenolic adhesives (e.g., for flooring agglomerates), or thermoplastic polymers such as polyurethanes (e.g., for softer surfacing materials).

The cork agglomerates may be produced with different densities depending on the targeted application: 200–300 kg m^−3^ for surfacing, partitioning and insulation applications, and higher densities up to 500 kg m^−3^ for flooring [6]. Cork agglomerates have a Young’s modulus of 7.4 MPa in compression and 17.4 MPa in tension [53]. The thermal conductivity coefficient is in the range of 0.06–0.10 W m^−1^ K^−1^ [15,54], the average specific heat capacity is 3370 J kg^−1^ K^−1^ and the thermal diffusivity 1.04107 m^2^ s^−1^ [55]. The cork composites have higher density in comparison to the natural cork due to the resin component and the compression during the agglomeration process that causes some densification of the cork cells with corrugation and collapse (Figure 4B). The properties of cork composites depend on their density and granulometry of the cork granules, the type and proportion of the binding resin, and on the production conditions. However, as can be expected, they resemble those of cork since the aim is to obtain a product similar to natural cork but allowing more complex shapes and larger dimensions [6].

### 3.3. Expanded Cork Agglomerates

**Figure 5:**
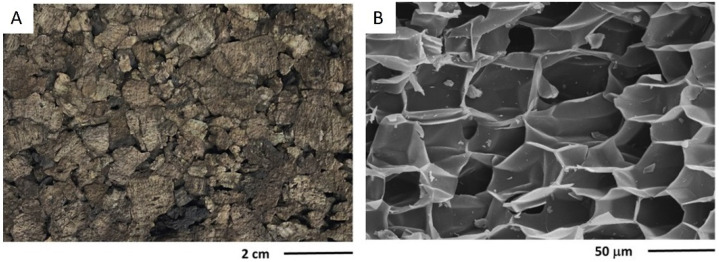
Visual aspects of an expanded cork agglomerate with 120 kg m^−3^ density and 0.5–2 cm granules (**A**), as well as its cellular structure observed by scanning electron microscopy (**B**). Photos by Helena Pereira.

Expanded cork agglomerates are produced with the most undervalued cork raw materials, using granules with dimensions in the range of 3 mm to over 22 mm that are self-bonded, i.e., without an external binder. Figure 5A exemplifies the macroscopic aspect of an expanded corkboard showing the dark-coloured granules that are bound together but with significant intergranular voids. Expanded cork agglomerates are applied mainly in thermal insulation, acoustical absorption, and vibrational damping. They are produced in blocks by heating the cork granules in closed autoclaves with superheated steam at 300–350 °C and 40 kPa, for 17 to 30 min. Under these conditions, there is a substantial structural and chemical alteration of cork: the cells expand, cell walls stretch and decrease in thickness, and the cell shape changes from prismatic to a more balloon-type with a cell volume increase of over 100% [56]. Figure 5B exemplifies the cellular features of the expanded cork granules, showing the significant expansion of the cork cells when compared to cork (Figure 2), thereby appearing as stretched cell walls without undulations.

There is a thermochemical degradation of the cork chemical components, with the release of gaseous by-products that reduce the cork mass up to 30%, and of non-volatile compounds that act as natural adhesives between the granules to form the agglomerate [40,56]. These agglomerates are also called black agglomerates due to their dark colour induced by the high temperatures associated with this process (Figure 4B).

The manufacturing process allows producing expanded cork with different densities that may range from below 100 to 300 kg m^−3^, depending on the range of applications. For thermal insulation, the density frequently ranges from 90 to 110 kg m^−3^ (standard density), while for acoustic insulation, the value is about 90 kg m^−3^ and vibrational layers have the highest densities. The granulometry also depends on the targeted application, e.g., from 3 to 10 mm for acoustic applications and from 5 to 22 mm for thermal insulation. The compaction between the cork granules in the expanded agglomerates varies with their density, and in the less-dense expanded agglomerates, there are extensive voids between granules, e.g., a 130 kg m^−3^ corkboard has 16% of voids [57].

The bonding between the cork granules in the expanded agglomerates is weaker than in the cork composites, although it is strong enough to allow the physical integrity of the boards and their handling during application. Therefore, the mechanical resistance properties of the expanded cork agglomerates are very much below those of the cork composites and of cork (Table 2 and Table 3). On the contrary, the insulation properties are better than those of cork. The expanded corkboards maintain their physical properties in very low temperatures, therefore performing better than other insulators [33]. Expanded cork agglomerates are also considered as a material of retarded combustion and, under fire conditions, they do not release toxic substances, as may occur with alternative materials [32].

The expanded cork granules are similar to cork regarding hydrophobicity and low water absorption.

Regarding acoustic absorption, the expanded cork agglomerate absorbs part of the total incident sound energy, thus reducing the intensity of the reflected sound, which is favoured by its irregular surface with many cavities that increase the reflection of the sound waves, resulting in a loss of energy for each wave [58].

## 4. Cork Properties Relevant for Façades and Prospective Research

As external cladding of façades, cork products have some advantageous properties by themselves and also compare favourably to conventional and current materials. The properties of cork and expanded cork agglomerates, as summarized in Table 1, Table 2 and Table 3 were critically evaluated in relation to the requirements of buildings and their potential performance as cork façades. This reasoning approach allows the following considerations:

- Low density: the lightness of cork allows for reduction in the structural requirements of the façades and decreases the risks in case of façade fracturing and falling.
- Hydrophobicity and low moisture absorption: the low affinity to water regarding surface adherence and water absorption is important to withstand rain and high atmospheric humidity that potentiate chemical and biological degradation.
- Physical and chemical stability under high and very low temperatures: this characteristic is important for external long-term exposure to high summer temperatures or to prolonged freezing temperatures.
- Fire behaviour: cork starts to degrade significantly only at temperatures above 300 °C, and, while on fire, it retains its cellular architecture and does not emit toxic gases, contrary to what happens to synthetic materials that lose their physical integrity at rather low temperatures and emit noxious gases.
- Thermal insulation properties: the low conductivity coefficient allows cork to be an efficient protection barrier against temperature differences.
- Acoustic insulation properties: the absorption of sound waves is an advantageous characteristic that favours the comfort of the built environment.
- Weathering resistance: under external exposure, cork maintains its physical and chemical integrity and only shows surface colour bleaching.
- Durability: overall, cork has a significant durability, with chemical and biological inertia.
- High environmental value due to its biological origin, the sustainable forest exploitation process, the ecological richness of cork oak forests, and the full resource approach taken by the industry.

Overall, cork products, including the raw cork, cork composites and expanded cork agglomerates, share these performance characteristics, with some differences related to their specific features.

The review synthesis on cork as a material that has been presented so far clearly shows that a large amount of research has been carried out. However, some knowledge gaps were found when considering the use of cork in external environments that will advise targeted research. The following examples highlight some of the prospective research areas:

- The weathering behaviour is certainly one area, with scarce information currently limited to one-year exposure of cork [48], and long-duration experiments are certainly needed, as well as testing of expanded corkboards in outdoor conditions. It will be also interesting to know the performance of cork under more challenging exposure conditions, e.g., saline environments, polluted industrial premises, high moisture or acid rain.
- The fire behaviour of cork and of cork products has been comparatively less studied, namely regarding the fire impact on the materials and on their protective function. Although it is known that the cork layer protects the trees during forest fires [59,60,61], and the thermal behaviour of cork and cork components have been researched [46], more knowledge is needed on the effect of very high temperatures on the structure and chemistry of cork, and on their dynamics across the cork layer.
- Further research should be carried out regarding new materials including cork, some of which are already in use, e.g., paints and cement layers containing cork particles, as well as several proposed composite materials that include other materials, e.g., plastic, stone, wood, fibrous biomass [62,63,64,65].

## 5. Architectural Examples of Cork in Façades

In early times, in the regions where cork oaks were present, cork was used by the local populations for construction or cladding of walls and roofs in dwellings and refuges, taking advantage of its insulation and impermeability properties as well as lightness and durability. Wilton and Howland [66] made an historical overview of cork in construction, gathering the records that exist in the literature, since little physical evidence remains today. For instance, in his *Natural History*, Pliny the Elder (23–79 A.D.) refers to roof coverings of houses similar to what is reported later in the sixteenth century, by Duarte de Armas in his *Book of Fortresses*, in which he described cork plank roofs in fortified settlements. Cork was used as strips or pieces of virgin cork and of planks of reproduction cork, applied externally as facing and lining, as well as inside thick earth walls [67]. In these early times, cork was used for construction only in regions with cork oaks and as a local raw material provided by the stripping of the tree stem, mostly applied in modest houses, and it is not strange that few examples have survived.

**Figure 6:**
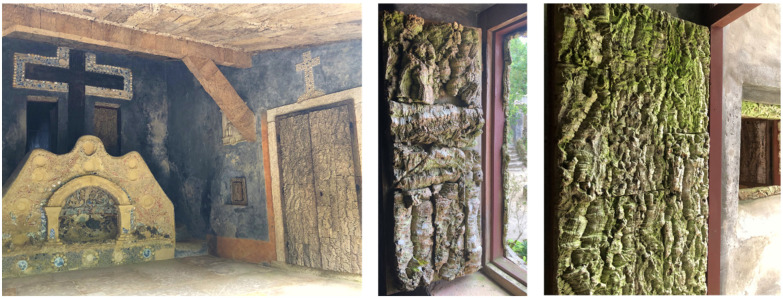
Images of cork use in the Convento dos Capuchos (Serra de Sintra, Portugal) as outdoor door covering (left) and as internal door frames (right). The cork used is virgin cork. Photos by Helena Pereira.

**Figure 7:**
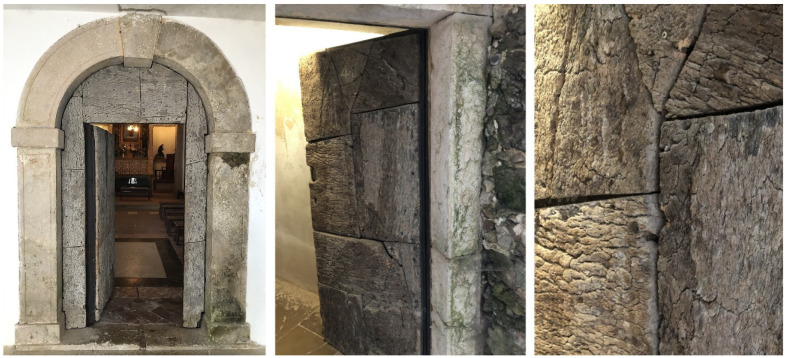
Images of cork use in the Convento da Arrábida (Serra da Arrábida, Portugal) as outdoor door covering to the chapel (left) using assembled cork planks of reproduction cork (right). Photos by Helena Pereira.

One existing example from the sixteenth century is the Convento dos Capuchos (Serra de Sintra, Portugal), a small Franciscan monastery built in a harsh environment around huge rocks, where virgin cork was conspicuously used as construction material (Figure 6). This monastery was founded in 1560 by friars coming from the Convento da Arrábida (Serra da Arrábida, Setúbal, Portugal), where cork was also applied, although less extensively and in the form of reproduction planks (Figure 7). The monastery of Capuchos comprises a church and eight tiny cells showing the use of virgin cork as an architectural material, both in external door cladding as well as in internal applications of wall covering and door framing. The virgin cork contributed to the decorative design of the construction, with its rustic character resembling a rough-cut stone, but also to thermally insulate the rooms and to establish a humidity barrier, thereby giving some natural comfort in the cold and damp climate of this hilly slope.

Cork started to become a more important, and economically more valuable, raw material when the use of cork stoppers for glass bottles increased by the end of the seventeenth century, leading to the development of international trade and the manufacture of cork stoppers, which soon became industrially produced in large factories by the mid-nineteenth century. The large amount of cork by-products and waste generated by the production of stoppers was a driver for the development of cork composite products, where cork granules were combined with binders and made into several construction materials, e.g., tiles and boards used in interior floor coverings and wall panelling.

The discovery of the process to make expanded cork agglomerates by John Smith, who patented the Consolidated Cork product in 1891, led to an extensive application of this pure cork agglomerate in construction as an insulation material in homes and in cold storage. Many examples exist in the USA and in Europe of the use of cork for the insulation of external walls in modern architecture, from architects such as Le Corbusier, Gropius, Alvar Aalto and Frank Lloyd Wright [66]. In these buildings, the expanded cork was used as an internal element in the façades.

**Figure 8:**
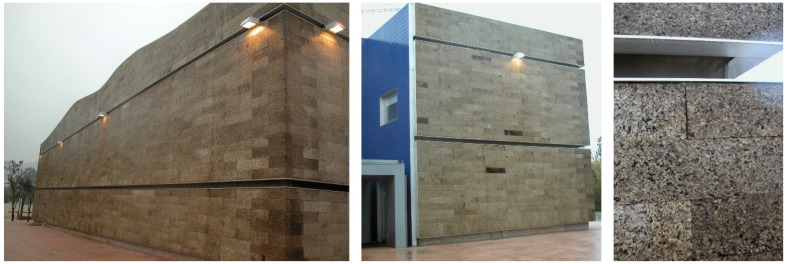
The Portugal Pavilion at Expo 2000 in Hannover, Germany, designed by architects Álvaro Siza and Eduardo Souto de Moura with façades covered by uncoated expanded corkboards, reconstructed in a city park of Coimbra facing the river Mondego, photographed in 2004 after two years of outside exposure after its 2002 construction. The NE façade is shown (left), as well as details of the uncoated expanded corkboards that were used. Photos by Helena Pereira.

**Figure 9:**
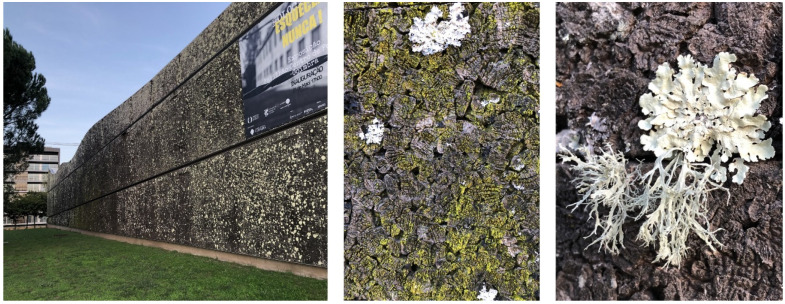
Photographs taken in 2021 of the reconstructed Portugal Pavilion from Expo 2000 in Hannover, in a city park of Coimbra facing the river Mondego after almost 20 years of outdoor exposure. The NE façade is shown (left), as well as details of the uncoated expanded corkboards. Photos by Helena Pereira.

Only very recently have architects dared to use the expanded cork agglomerates as façade cladding, thereby adding their unusual visual aspect to their properties and positive environmental profile. The first were the architects Álvaro Siza and Eduardo Souto de Moura, who designed the Portugal Pavilion for Expo 2000 in Hannover, with an external façade cladding of expanded cork agglomerate that partially covered the building. In the few months of the fair, the building and its dark-coloured expanded corkboards attracted considerable attention, and this innovative façade cladding confirmed its good performance. After the exhibition, the pavilion was reconstructed in 2002 in a city park of Coimbra, now serving as a municipal building for events and concerts (Figure 8). With two decades of outside exposure, it is now possible to observe the long-time performance of expanded corkboards. In 2004, with two years of outdoor exposure, the façades covered with the uncoated expanded cork agglomerate appeared as they were at the time of construction, only showing the brown chromatic dynamics of the different corkboards. In 2021, after almost two decades of outdoor exposure in the humid environment of its location near to the river Mondego, the NE façade shaded by nearby trees showed conspicuous signs of biological growth of moss and lichens, and the cork surface changed to a very dark colour (Figure 9). The use of standard low-density corkboards with an appreciable amount of intergranular voids and a rough cork surface (details of Figure 8 and Figure 9) allowed the entrapment of water and of solid materials that favoured biological growth. These facts suggest that an expanded corkboard with higher density i.e., without intergranular voids and a smoothed surface, will avoid biological growth and adherence of dust and other contaminations. Although the cost of such material is higher, the maintenance costs will be significantly lower, and the façade aesthetics will be maintained; thereby it is recommended for long-duration façade coverings.

**Figure 10:**
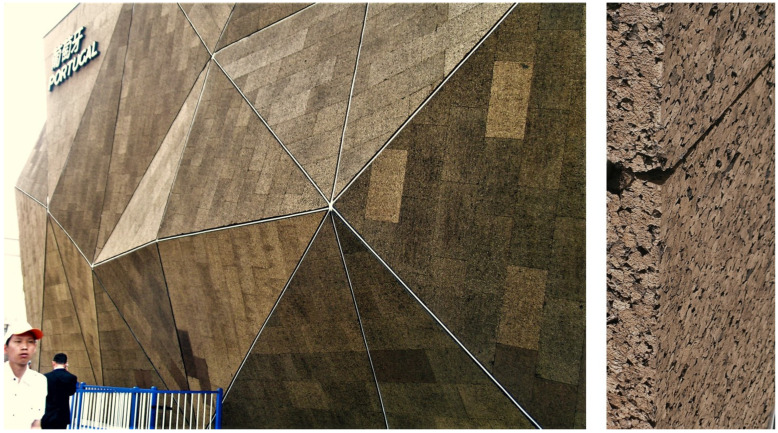
The Portugal Pavilion at Expo 2010, Shanghai, China, designed by architect Carlos Couto, completely covered by uncoated expanded corkboards, and application detail of the used corkboards. Photos by Helena Pereira.

In 2010, at Expo 2010 in Shanghai, China, the Portuguese pavilion again featured uncoated expanded corkboards as external cladding. The pavilion, designed by architect Carlos Couto, had a striking appearance as a massive polyhedral solid without openings, completely covered by a total of 3640 m^2^ of expanded insulation corkboards, with their typical dark brown colour (Figure 10). The pavilion attracted a lot of curiosity, registering over 5 million visitors, and won a gold medal in the Design category awarded by the International Exhibition Bureau. The cork façades showed the typical chromatic variation of the different boards around the dark brown colour, and the overall performance during the four months of the exhibition was excellent. The insulation boards had standard density, i.e., showing the conspicuous granular structure with some apparent voids, which triggered attempts from some visitors to detach cork granules! As previously discussed, while this cladding is perfect for short-term exposure, over the years, it may incur accumulation of water and inert materials and biological growth.

A temporary exhibition that also attracted international attention was an installation by Herzog & de Meuron and Ai Weiwei at the Serpentine Gallery’s Summer Pavilion in the Kensington Gardens (London, UK), in 2012, featuring a circular complex multi-level structure for which 80 m^3^ of expanded insulation corkboards were carved into stairs, platforms, benches and stools. The Serpentine Pavilion 2012 is currently in a private collection relocated to the Surrey garden of Indian steel magnates Usha and Lakshmi Mittal.

After these well-known works with outdoor application of expanded corkboards, projects using cork façades increased significantly, not only in the cork-producing countries but also elsewhere, including various projects for small-scale cabins or booths, for family houses, as well as for larger buildings, e.g., wineries, hotels, schools and offices. Also, innovative designs with expanded corkboards have been proposed, such as a self-sustained dome [68] or for temporary shelters [69]. The largest world producer of expanded corkboards, Amorim, keeps a platform with most of the constructed designs with cork façades [70].

A few examples are reported here of projects of different types that have been built and have received several awards for design and architecture:

- The 3-storey HIGO library and offices, designed by Makoto Nakayama in 2014, in Sapporo, Japan, has a façade covered with uncoated expanded corkboards [71].
- The Corkscrew house, designed by Rundzwei Architekten, was constructed in 2018, in Berlin (Germany), with all façades covered by uncoated expanded cork agglomerate [72].
- The Corkhouse by Mathew Barnett Howland, Dido Milne and Oliver Wilton, built in 2019 in Berkshire (UK), that uses solid load-bearing blocks of expanded cork agglomerate as walls and roof, with a concept of self-build construction designed for disassembly. The project was conceived as a kit of parts, with off-site prefabricated components that are assembled by hand on-site without mortar or glue [73].
- The Float was designed by the architecture firm Studio RAP as a floating home in a canal in the centre of Leiden (The Netherlands) in 2021, which features an external cladding with uncoated high-density expanded corkboard that was molded by CNC cutting, and an internal layer of low-density expanded cork [74].

**Figure 11:**
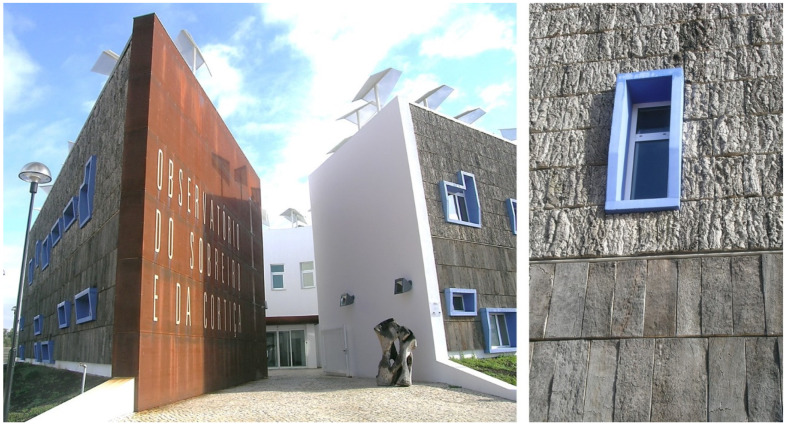
The Observatory for Cork Oak and Cork, designed by architect Manuel Couceiro, built in 2009 in Coruche, Portugal, with the façades covered by planks of reproduction cork on the lower part, and by pieces of virgin cork in the upper part of the façades. Photos by Helena Pereira.

Another example of cork façades but not with the expanded cork agglomerate is the Observatory for Cork Oak and Cork, designed by architect Manuel Couceiro, built in 2009 in Coruche, Portugal, and owned by the municipality, with the façades covered by planks of reproduction cork on the lower part, and by pieces of virgin cork in the upper part of the façades (Figure 11). The concept was of making a parallel to the cork oak landscape with trees that have, in the lower part of the stem, reproduction cork while the upper stem maintains its virgin cork. In this case, the typical colour bleaching that is noticed in the other designs do not apply here to a significant extent since the virgin cork has already undergone the full colour change to a greyish tonality while in the tree, and the lignocellulosic covering of the reproduction cork planks also acquired the typical dark grey tonality during their nine years in the tree. The performance of the façades has been excellent during the 15 years since construction.

## 6. Concluding Remarks

Cork is a natural material of biological origin and with a sustainable production that make it a green and eco-friendly material for construction, while its set of properties allows for outstanding performance by combining lightness, thermal and acoustic insulation, very low permeability to water and vapor, surface hydrophobicity, chemical inertia and durability.

Cork has attracted the attention of well-known architects for outdoor application, thereby triggering the spread of an innovative use of cork as cladding of façades, with numerous projects of different typology constructed in different countries. Research on cork and reviews on its properties have been targeted towards the most used applications such as closures, surfacing and interior insulation layers. This is the first assembly of cork properties and a critical performance appraisal for the use of cork in outdoor applications, namely as building façades, acknowledging the lack of studies on weathering of cork and the impact of environmental conditions on its structure, chemistry and properties.

Expanded cork agglomerates have been the most-selected materials for building façades, and the existing contemporary examples empirically allow following its performance along the years, which has been, overall, excellent. Incorporation of the colour dynamics of the corkboards over time in the design should be taken into account, as well as the importance of using a dense expanded corkboard without intergranular voids whenever longtime duration is envisaged. Research studies should be targeted on the structure, chemistry and performance of expanded cork under long outdoor exposure in various climatic conditions. The development of new creative designs and experimenting with other cork-containing materials certainly will enrich our knowledge on cork façades and support their enhanced use.

## Figures and Tables

**Table 2:** Properties of expanded cork agglomerates for thermal insulation with 100–160 kg m^−3^ density.

Property	Value	References
Thermal conductivity coefficient (θm = 23 °C) dry after one wetting/drying cycle λ_(23)_	0.039–0.045 W m^−1^ K^−1^ 0.041–0.054 W m^−1^ K^−1^ 0.042–0.056 W m^−1^ K^−1^	[9,32]
Specific heat (to 20 °C)	1.7–1.8 kJ kg^−1^ K^−1^	[32]
Thermal expansion coefficient (20 °C)	40 × 10^−6^	[33]
Thermal diffusion	0.18–0.20 × 10^−6^ m^2^ s^−1^	[32]
Water vapour transmission properties vapour transmission rate water vapour permeance water vapour resistance water vapour permeability	455.54–490.3 mg h^−1^ m^−2^ 0.33 mg m−^2^ h^−1^ Pa^−1^ 3.09 m^2^ h Pa mg^−1^ 0.01 mg m^−1^ h^−1^ Pa^−1^	[9,32]
Maximum pressure in elastic conditions	50 kPa	[32]
Modulus of elasticity (compression)	1900–2800 kPa	[32]
Modulus of rupture (bending strength)	140–200 kPa	[9,32]
Poisson coefficient	0–0.02	[32]
Tensile strength perpendicular to faces Dry condition Wet condition (for 28 days)	67.8–73.2 kPa 64.2–68.8 kPa	[9,32]
Dimensional stability length width thickness	0.3–0.31% 0.3–030% 0.4–0.5%	[9,32]
Oxygen index	26%	[32]
Tension deformation at 10% (compression)	150–180 kPa	[32]
Temperature deformation (80 °C)	1.4 to 2.4% (thickness)	[32]

**Table 3:** Properties of expanded cork agglomerates for acoustic applications, with 90–110 kg m^−3^ density.

Property	Value	References
Sound absorption coefficient (500–1500 c/s)	0.33–0.8	[32]
Thermal conductivity coefficient (θm = 23) dry after one wetting/drying cycle λ_(23/50)_	0.037–0.042 W m^−1^ K^−1^ 0.039–0.044 W m^−1^ K^−1^	[9,32]
Water vapour transmission properties vapour transmission rate water vapour permeance water vapour resistance water vapour permeability	813.64–889.36 mg h^−1^ m^−2^ 0.58 mg m−^2^ h^−1^ Pa^−1^ 1.73 m^2^ h Pa mg^−1^ 0.04 mg m^−1^ h^−1^ Pa^−1^	[9,32]
Water absorption (immersion) (capillarity)	9.2% 1.9%	[32]
Modulus of rupture (bending strength)	87.6–160 kPa	[9,32]
Tensile strength perpendicular to faces dry condition wet condition (for 7 days) wet condition (for 28 days)	60.7–69.1 kPa 58.5- 67.1 kPa 44.4–49.0 kPa	[9,32]
Compressive stresses at 10% strain Thickness 50 mm Thickness 70 mm	154–160.1 kPa 131–134 kPa	[9]
Shear strength	55–58 kPa	[9]
Compressive force	0.63–0.76 kPa	[9]
Deformation under specified compressive load at 23 ± 5 °C for 48 ± 1 h at 80 ± 1 °C for 48 ± 1 h	0.302–0.315% 6.844–7.51%	[9]
Dimensional variation from 32–66 °C, 90–0% HR	0.4%	[9,32]

## Data Availability

Data are contained within the article.
